# Association of atrial fibrillation susceptibility genes, atrial fibrillation phenotypes and response to catheter ablation: a gene-based analysis of GWAS data

**DOI:** 10.1186/s12967-017-1170-3

**Published:** 2017-04-05

**Authors:** Daniela Husser, Petra Büttner, Laura Ueberham, Borislav Dinov, Philipp Sommer, Arash Arya, Gerhard Hindricks, Andreas Bollmann

**Affiliations:** Department of Electrophysiology, Heart Center Leipzig, Leipzig University, Strümpellstr. 39, 04289 Leipzig, Germany

**Keywords:** Atrial fibrillation, Catheter ablation, Genome wide association study, Gene-based analysis

## Abstract

**Background:**

Previous studies have suggested *PITX2, KCNN3* and *ZFHX3* as atrial fibrillation (AF) susceptibility genes. Single common genetic polymorphisms of those genes have been linked with AF phenotypes and rhythm outcome of AF catheter ablation although their mechanisms remain elusive. New gene-based association tests may help clarifying genotype–phenotype correlations. Therefore, we hypothesized that *PITX2, KCNN3* and *ZFHX3* associate with left atrial enlargement and persistent AF and subsequently with ablation outcome.

**Methods and results:**

Samples from 660 patients with paroxysmal (n = 370) or persistent AF (n = 290) undergoing AF catheter ablation were genotyped for ~1,000,000 SNPs. Gene-based association was investigated using two different gene-based association tests (VEGAS, minSNP). Among the three candidate genes, only *ZFHX3* associated with left atrial dilatation and AF recurrence after catheter ablation.

**Conclusion:**

This study suggests a contribution of *ZFHX3* to AF remodeling and response to therapy. Future and larger studies are necessary to replicate and apply these findings with an emphasis on designing AF pathophysiology-based multi-locus risk scores.

## Background

Single nucleotide polymorphisms (SNPs) at chromosomes 4q25 (*PITX2*) [1, 2], 16q22 (*ZFHX3*) [3], and 1q21 (*KCNN3*) [4] have been shown to associate with atrial fibrillation (AF) in different genome-wide association studies (GWAS).

Catheter ablation is an established AF treatment, but arrhythmia recurrence occurs in up to 50% of patients within 1 year after ablation [5]. Previous work has consistently identified left atrial enlargement and persistent AF as clinical predictors for ablation success [6]. Recent candidate-gene studies have linked common genetic variants with intermediate AF phenotypes such as left atrial fibrosis [7] as well as rhythm outcome after AF ablation [8–10].

However, implicated genetic variants extend over broad genomic distances, often spanning several thousands of bases raising the possibility that those loci may contain multiple independent susceptibility signals. In addition, linkage disequilibrium (LD) structure and genotyping coverage may impact on GWAS results when only analyzing single SNPs. Consequently, gene-based association tests have been introduced that are well-suited to identify genes that may increase susceptibility to complex diseases, phenotypes and response to therapy. They have been designed to detect genes that are genome-wide significant, but where no single SNP effect is large enough to be genome-wide significant by univariate tests.

Here, for the first time, we apply gene-based analysis of GWAS data to test the hypothesis that AF candidate genes *PITX2, KCNN3* and *ZFHX3* associate with left atrial enlargement and persistent AF and subsequently with arrhythmia recurrence following AF catheter ablation.

## Methods

### Patients

Six hundred-and-sixty patients with non-valvular AF undergoing de-novo radiofrequency AF catheter ablation between 2008 and 2013 were enrolled in the Leipzig Heart Center AF ablation registry. Paroxysmal AF was defined as self-terminating episodes of AF within 7 days after onset documented by ECG or an ambulatory ECG monitor. Persistent AF was defined as an AF episode either lasting longer than 7 days or requiring drug or direct current cardioversion for termination.

Transthoracic and transesophageal echocardiography (TEE) was performed prior to catheter ablation. Left atrial diameter (LAD) and left ventricular ejection fraction were determined using standard measurements and a left atrial thrombus was excluded. All class I or III antiarrhythmic medications with the exception of amiodarone were discontinued at least 5 half-lives before the procedure.

### AF catheter ablation and follow-up

Left atrial catheter ablation was performed using a previously described approach [8, 11]. In brief, patients were studied under deep propofol sedation with continuous invasive monitoring of arterial blood pressure and oxygen saturation. Non-fluoroscopic 3D catheter orientation, CT image integration, and tagging of the ablation sites were performed using Ensite NavX, Ensite Velocity (St. Jude Medical, St. Paul, MN, USA) or CARTO 3 (Biosense Webster, Diamond Bar, CA, USA). Trans-septal access and catheter navigation were performed with a steerable sheath (Agilis, St. Jude Medical, St. Paul, MN, USA). Patients presenting with AF at the beginning of the procedure were electrically cardioverted and ablation was performed during sinus rhythm (i.e. AF termination by ablation was not attempted). In all patients circumferential left atrial ablation lines were placed around the antrum of the ipsilateral pulmonary veins (irrigated tip catheter, pre-selected tip temperature of 48 °C, and maximum power of 20–40 W). In patients with persistent AF, linear lesions were added at the left atrial roof, the basal posterior wall and the left atrial isthmus or in low voltage areas (LVA) until 2011. In patients recruited between 2011 and 2013, electro-anatomical voltage mapping to characterize LVA defined as potentials below 0.5 mV was performed as previously described [11]. After circumferential line placement, voltage and pace mapping along the ablation line were used to identify and close gaps. The isolation of all pulmonary veins with bidirectional block was verified with a multipolar circular mapping catheter and was defined as the procedural endpoint. Burst pacing from coronary sinus (down to 200 ms) was performed at the end of the procedure. If sustained AF was induced, patients were electrically cardioverted and no additional ablation was performed. If atrial tachycardia was induced, those were mapped and ablated.

After ablation, class I and III antiarrhythmic drugs were not reinitiated. Oral anticoagulation was prescribed for 6 months, and proton pump inhibitors were added for 4 weeks. All patients were followed in the outpatient clinic for 12 months after the ablation. During this follow-up period, 7-day Holter ECG recordings were performed 3, 6 and 12 months after the ablation. Additional ECGs and Holter ECG recordings were obtained when patients’ symptoms were suggestive of AF. AF recurrence was defined as a documented AF episode lasting longer than 30 s between 3 and 12 months after the ablation (thus, including a 3-month “blanking period”). All patients with sustained early recurring AF underwent direct cardioversion. Additional drug administration was left to the discretion of the treating physician.

### Sample processing

Blood samples were obtained in EDTA test tubes in fasting state prior ablation. Genomic DNA was isolated using a commercial kit according to the manufacturer’s recommendations (PeqLab, Erlangen, Germany). Genotyping was performed using HumanOmniExpressExome-8-v1.2 arrays comprising about one million single nucleotide polymorphisms (SNPs) according to established protocols (Illumina, San Diego, US).

### Data analysis and statistics

Raw data was compiled using GenomeStudio (Illumina) software and exported to PLINK GWAs analysis package [12]. Using PLINK tool set the data was tested for consistency. Samples with a call rate <95% were excluded. Single SNPs had to meet the following criteria: minor allele frequencies (MAF) > 0.01, call rate >95%, Hardy–Weinberg equilibrium (HWE) significance threshold >0.0001. Otherwise they were excluded from further analysis.

Association of genotypes with LAD was detected using linear regression with adjustment for age, gender, body mass index and AF type. Association of genotypes with AF type (persistent AF) and arrhythmia recurrence was detected using logistic regression analysis with adjustment for age and gender.

Illumina’s exome arrays contain specific “exm-SNPs” which were assigned to their corresponding dbSNP rs IDs prior further analysis.

We used two different gene-based tests (minSNP, VEGAS [versatile gene-based association study]) whose performance depend on different genetic architectures (multiple independent signals versus single signal in a gene, size of gene, patterns of LD in the gene and so on) [13]. minSNP calculates a gene-based *P*-value either directly from a parametric distribution or by using the permuted *P*-value of the best individual SNP association within the given gene. minSNP computes single-SNP F-statistics for each SNP within a gene and uses the best F-statistic within that gene as its test statistic, which is then converted to a *P*-value with gene-based permutations to correct for gene size [13]. VEGAS uses the sum of *X* ^2^ for an individual SNP to generate a test statistic suggested for the gene. The *P*-value of the gene is then computed after accounting for LD and the number of SNPs in each gene [14].

SNPs within 200 kB of the candidate genes were selected for inclusion. This range was chosen based on previous studies implicating common 4q25 variants 195 kB upstream of *PITX2* in AF susceptibility and response to therapy.

We applied a two-stage analysis plan. First, we identified consistently associated genes with LAD and AF type. For this step, a Bonferroni correction was applied to account for the analysis of three genes and two phenotypes (*P*-value less than 0.05/6 = 0.0083).

Second, association of those identified gene(s) with AF ablation outcome was tested. For this analysis, we divided the cohort into two data sets based on the ablation period and strategy (data set 1: n = 496, 2008–2011, pulmonary vein isolation ± linear lesions; data set 2: n = 164, 2011–2013, pulmonary vein isolation ± voltage-guided substrate modification) and performed a meta-analysis on individual data for the entire cohort.

eQTL analyses of significant SNP(s) were performed using the publicly available genotype-tissue expression portal (GTEx) of the Broad Institute of Harvard and MIT (GTex, Broad Institute, Boston, MA, USA; http://www.gtexportal.org/home/).

Clinical variables are presented as mean ± one standard deviation or percentages. They were compared between patients with and without AF recurrence using Chi square or Student’s t-test.

## Results

### Patient characteristics

The study population included 660 patients with a history of paroxysmal (n = 370) or persistent AF (n = 290, Table 1). LAD was available in 538 of those data sets and could not be analyzed due to insufficient echo conditions or performance of TEE only in the remaining patients. LAD was significantly larger in patients with persistent AF (41 ± 5 vs. 45 ± 6 mm, *P* < 1.0E−3) compared to patients with paroxysmal AF. Both data sets were different with respect to gender, AF type, LAD and LVEF but AF recurrence rate was identical (Table 1).

**Table 1:** Patient characteristics

	Total (n = 660)	Data set 1 (n = 496)	Data set 2 (n = 164)	*P*-value
Age (years)	60 ± 10	59 ± 10	61 ± 10	ns
Male gender (%)	68	66	75	2.1E−02
Body mass index (kg/m^2^)	28.9 ± 4.6	28.9 ± 4.5	29.0 ± 4.7	ns
Idiopathic AF (%)	14	13	15	ns
Persistent AF (%)	44	38	61	3.3E−07
LAD (mm)	43 ± 6	42 ± 6	44 ± 6	2.0E−03
LVEF (%)	59 ± 10	60 ± 9	57 ± 10	1.0E−03

AF recurrence between 3 and 12 months was observed in 48% (Table 2) and was associated with LAD (OR 1.05 per mm increase, 95% CI 1.02–1.08, *P* = 1.0E−3) and persistent AF (OR 2.1; 95% CI 1.567–2.931, *P* = 2.0E−6) in multivariate analysis.

**Table 2:** Patient characteristics in patients with and without AF recurrence

	AF recurrence (n = 318)	No recurrence (n = 341)	*P*-value
Age (years)	61 ± 10	59 ± 10	7.55E−03
Male gender (%)	70	67	ns
Body mass index (kg/m^2^)	29.2 ± 4.6	28.7 ± 4.5	ns
Persistent AF (%)	53	35	1.00E−06
LAD (mm)	44 ± 6	42 ± 6	5.42E−05
LVEF (%)	58 ± 10	59 ± 9	ns

One patient was lost to follow-up

Genotyping call rate in all subjects was >95% except in three samples (<85%) that were excluded from further analysis.

### Gene-based associations

Results from the gene-based tests for association between AF candidate genes and AF phenotypes are summarized in Table 3. *KCNN3* associated with LAD and AF type but failed to reach pre-defined significance threshold while *PITX2* was not consistently associated with any of the phenotypes. *ZFHX3* was significantly associated with LAD in both tools and AF type in VEGAS but not in minSNP.

**Table 3:** Gene-based association results

	LAD		AF type	
	VEGAS	minSNP	VEGAS	minSNP
*KCNN3*	0.0153	0.0396	<0.00001	0.0348
*PITX2*	0.0083	ns	ns	ns
*ZFHX3*	0.0001	0.0043	0.0001	ns

**Fig. 1:**
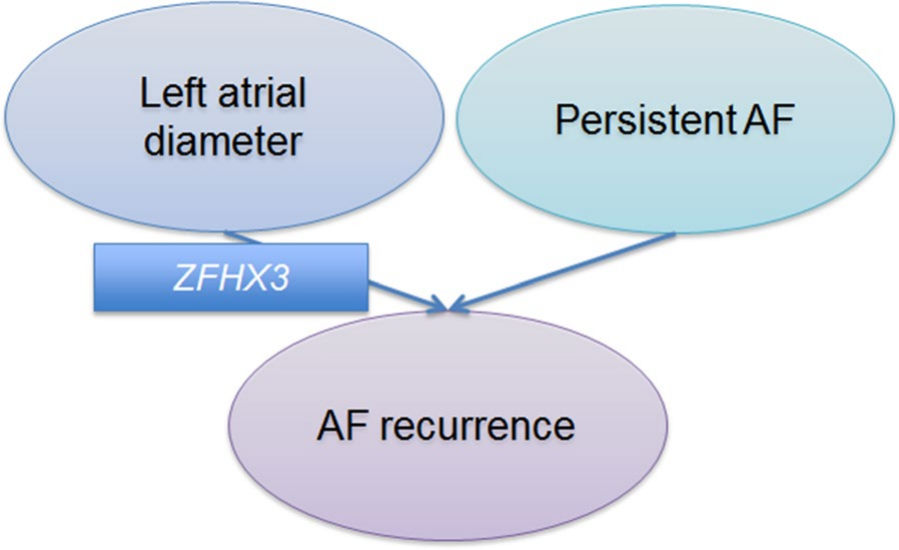
Left atrial enlargement and persistent AF are associated with rhythm outcome of AF catheter ablation, but whether or not they are associated with AF susceptibility genes is unknown. In this study, we found *ZFHX3* to be associated with left atrial diameter and, in turn, with AF recurrence

Therefore, association between *ZFHX3* and AF recurrence was assessed (Fig. 1). *ZFHX3* associated with AF recurrence in both data sets and subsequently in the entire cohort (*P* = 0.001 in VEGAS and *P* = 0.0288 in minSNP).

**Fig. 2:**
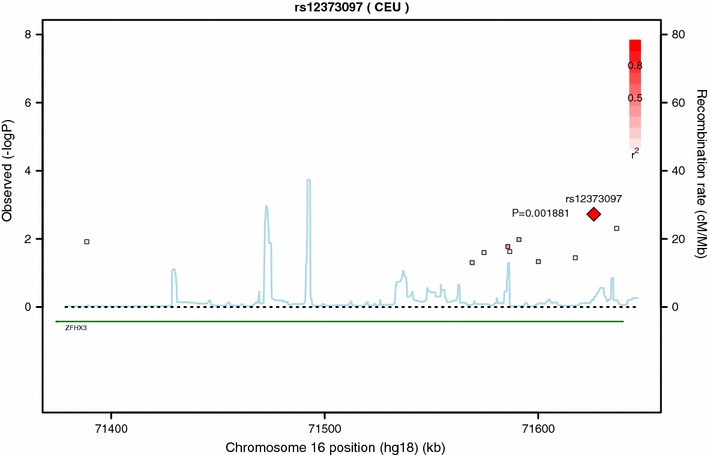
Regional association plot for all significant *ZFHX3* SNPs and response to AF ablation. The plot was created using GWAS association data that served as the input for gene-based testing. The *x-axis* represents the distribution of SNPs across *ZFHX3* while the *y-axis* represents the −log_10_ of the *P*-value of each SNP in the gene. The colors indicate the *r* ^2^ between the SNP with the lowest *P*-value and all the other SNPs. The *light blue* trace indicates recombination hotspots

By accessing the publicly available GTEx, a significant association between the top *ZFHX3* SNP (Fig. 2), rs12373097 (OR for LRAF 1.57, 95% CI 1.18–2.08, *P* = 0.002), with a higher expression of *ZFHX3* in fibroblasts (*P* = 8.0E−8) was found. This SNP had a minor allele frequency of 20.3 with 32% of patients being heterozygous and 4% being homozygous.

## Discussion

### Main findings

This study is the first to explore the association of AF candidate genes and AF remodeling-associated phenotypes such as left atrial enlargement and AF type as well as response to AF catheter ablation using gene-based analysis of GWAS data. *ZFHX3* was associated with left atrial diameter and ablation outcome in two different gene-based tests.

### Association between genotype, AF phenotypes and AF ablation outcomes

Different AF susceptibility alleles have been associated with non-pulmonary vein triggers and left atrial scar [7]. Interestingly, of those SNPs, one *ZFHX3* SNP (rs7193343) was the only genetic variant that contributed to increased risk of both phenotypes. Somewhat conflicting results have been obtained with left atrial parameters. There were no associations between candidate SNPs and several left atrial measures in the Framingham cohort. However, after excluding prevalent AF, a single SNP (rs2200733) in proximity to *PITX2* was associated with left atrial volume following adjustment for body surface area. A second SNP (rs2106261) in proximity to the *ZFHX3* gene was related to LAD [15].

Common genetic variants have also been associated with ablation outcome. Of those, the 4q25 variants have been shown the strongest association in single-center studies and a recent meta-analysis, [8, 10] although results have not been consistent [16, 17].

In this study, we focused on gene-based tests and first identified an association between genotype and AF phenotypes LAD and AF type and then between genotype and AF ablation outcome. This was based on the fact that left atrial enlargement and AF persistence have consistently been linked with response to AF ablation outcome [6]. Our study revealed associations of the *ZFHX3* gene with left atrial dilatation and subsequently with rhythm outcome. This finding is supported by one previous study that identified the rs2106216 *ZFHX3* genetic polymorphism as independent predictor of a good ablation response [15] and strong biological evidence linking *ZFHX3* with AF and cardiac remodeling discussed below.

### *ZFHX3* and AF

*ZFHX3* encodes a cardiac transcription factor containing multiple homeodomains and zinc finger motifs and common *ZFHX3* variants have been shown to increase AF risk [3, 18]. Although their cardiac mechanisms underlying AF remain elusive, several gene effects have been demonstrated. For instance, *ZFHX3* is related to JAK/STAT signaling cascade that mediates the inflammatory process thereby contributing to electrical and structural remodeling of the atrium with inflammatory changes [19]. Interestingly, cross-regulation of gene expression of AF candidate genes has been suggested as molecular basis for gene–gene interactions [20]. The most significant *ZFHX3* SNP in our study, rs12373097, has been found by eQTL analysis to predict higher expression of *ZFHX3. ZFHX3* positively regulates expression of *PITX2* and both *ZFHX3* and *PITX2* positively regulate expression of *NPPA*. Atrial natriuretic peptide, the *NPPA* product, in turn, plays a key role in cardiac electrophysiology through APD shortening and calcium influx reduction, modulating the autonomic nervous system and regulating the function of cardiac ion channels [21]. In addition, both *ZFHX3* and *PITX2* impact on other downstream genes such as *TBX5, NKX2.5, KCNQ1* and *SCN1B* that play critical roles in cardiac electrophysiology and AF [20]. Of those, *TBX5* modulation has very recently been found to profoundly alter cardiac channel gene expression and to cause primary, spontaneous AF in mice [22].

Taken together, those and our findings implicate *ZFHX3* in several AF-remodeling associated processes that may underlie left atrial dilatation and AF progression and subsequently response to AF catheter ablation.

## Limitations

Our study is based on small sample size. However, it was based on the three strongest candidate genes that were selected for gene-based analysis of GWAS data a priori. In other words, although we used GWAS data, we applied a hypothesis-driven approach. Furthermore, we addressed this by using well-defined intermediate AF phenotypes that are known interrelated markers of AF ablation outcome. We only selected the gene that showed associations with intermediate AF phenotypes for further analysis of its association with ablation outcome. Finally, we required the genotype–phenotype correlation to be present in two different gene-based analysis tools accounting for different genetic architectures (multiple independent signals versus single signal in a gene, size of gene, patterns of LD).

Although we have used two data sets reflecting changes in patient characteristics and ablation strategies for ablation outcome, they came from the same institution with all inherent limitations for genetic studies but advantage of coherent patient selection, operators and follow-up. Despite those differences in clinical characteristics ablation outcome was identical. Nevertheless, ablation approaches in persistent AF are evolving and linear lesion sets have lessened in popularity due to no incremental benefit and even pro-arrhythmia. This may impact on type of arrhythmia recurrence and should be considered when assessing recurrence rates and comparing this to other studies.

This study does not contribute to the elaboration of molecular mechanisms which was beyond the scope of this study and did not address the question whether or not genotype impacts on proarrhythmic ablation effects or AF progression.

Finally, ablation outcome was assessed with serial clinical and prolonged Holter ECG monitoring which is in line with current recommendations but can nevertheless miss common asymptomatic AF episodes [23].

## Conclusions

These findings suggest a contribution of *ZFHX3* to AF remodeling and response to AF catheter ablation. Future and larger studies are necessary to replicate and apply these findings with a clinical emphasis on designing AF pathophysiology-based multi-locus risk scores.

## Abbreviations

AF: atrial fibrillation
GWAS: genome-wide association study/studies
LAD: left atrial diameter
SNP: single nucleotide polymorphism
